# Acute and chronic kidney disease in elderly patients with hip fracture: prevalence, risk factors and outcome with development and validation of a risk prediction model for acute kidney injury

**DOI:** 10.1186/s12882-017-0437-5

**Published:** 2017-01-14

**Authors:** Christine J. Porter, Iain K. Moppett, Irene Juurlink, Jessica Nightingale, Christopher G. Moran, Mark A. J. Devonald

**Affiliations:** 1 Renal and Transplant Unit, Nottingham University Hospitals NHS Trust, Nottingham, NG5 1PB UK; 2 Anaesthesia and Critical Care, Division of Clinical Neuroscience, University of Nottingham, Nottingham, NG7 2RD UK; 3 Information and Computer Technology, Nottingham University Hospitals NHS Trust, Nottingham, UK; 4 Department of Orthopaedic Trauma, Nottingham University Hospitals NHS Trust, Nottingham, UK; 5 Department of Anaesthesia, Nottingham University Hospitals NHS Trust, Nottingham, NG7 2UH UK

**Keywords:** Hip fracture, Acute kidney injury (AKI), KDIGO creatinine criteria, Hospital length of stay, Kidney function, Mortality, Postoperative AKI, Surgery, Surgical complication

## Abstract

**Background:**

Hip fracture is a common injury in older people with a high rate of postoperative morbidity and mortality. This patient group is also at high risk of acute kidney injury (AKI) and chronic kidney disease (CKD), but little is known of the impact of kidney disease on outcome following hip fracture.

**Methods:**

An observational cohort of consecutive patients with hip fracture in a large UK secondary care hospital. Predictive modelling of outcomes using development and validation datasets. Inclusion: all patients admitted with hip fracture with sufficient serum creatinine measurements to define acute kidney injury. Main outcome measures – development of acute kidney injury during admission; mortality (in hospital, 30-365 day and to follow-up); length of hospital stay.

**Results:**

Data were available for 2848 / 2959 consecutive admissions from 2007-2011; 776 (27.2%) male. Acute kidney injury occurs in 24%; development of acute kidney injury is independently associated with male sex (OR 1.48 (1.21 to 1.80), premorbid chronic kidney disease stage 3B or worse (OR 1.52 (1.19 to 1.93)), age (OR 3.4 (2.29 to 5.2) for >85 years) and greater than one major co-morbidities (OR 1.61 (1.34 to 1.93)). Acute kidney injury of any stage is associated with an increased hazard of death, and increased length of stay (Acute kidney injury: 19.1 (IQR 13 to 31) days; no acute kidney injury 15 (11 to 23) days). A simplified predictive model containing Age, CKD stage (3B-5), two or more comorbidities, and male sex had an area under the ROC curve of 0.63 (0.60 to 0.67).

**Conclusions:**

Acute kidney injury following hip fracture is common and associated with worse outcome and greater hospital length of stay. With the number of people experiencing hip fracture predicted to rise, recognition of risk factors and optimal perioperative management of acute kidney injury will become even more important.

## Background

Hip fractures are a common injury in the elderly in the United Kingdom [1] and worldwide [2]. These patients are at high risk for protracted length of stay, post-operative mortality and morbidity and reduction in quality of life [3]. Any patients who develop post-operative acute kidney injury (AKI) are also likely to have an increased length of stay and a greater likelihood of in hospital mortality and morbidity [4–9].

Little has been published on patients who have both problems, although The Scottish Intercollegiate Group hip fracture guidelines identified pre- and post-operative hydration and pre-admission renal dysfunction as areas requiring further attention and research [1]. Previous studies on hip fracture [10] and AKI [11] have provided patchy information based on admission serum creatinine (SCr) values or small populations. Current concepts in renal injury highlight increasing age, pre-existing chronic kidney disease, male gender, diabetes mellitus, heart failure and surgery as major risk factors for AKI [12–14]. However, in the hip fracture population, these may be relatively non-sensitive and non-specific risk factors since two thirds of these patients are aged over 80 and co-morbidity, including chronic kidney disease (CKD), is common at admission. Relatively small changes in SCr can predict or indeed define AKI. These changes may be missed or discounted in this population, particularly in the early stages of AKI. Although many underlying risk factors for AKI may not be modifiable in this cohort, an understanding of the risks of developing AKI, and the consequences for the patient, may be of use for the multidisciplinary team caring for these patients.

The aim of this study was to describe the renal morbidity of the hip fracture population and its association with short and long-term mortality and to identify key risk factors for AKI within this population. We also wished to use these data to develop and validate a risk prediction model for development of AKI following hip fracture.

## Methods

Nottingham University Hospitals NHS Trust has two substantial clinical databases developed by the Departments of Trauma and Orthopaedic Surgery and Renal and Transplantation. The Nottingham Hip Fracture database has been described previously [15, 16] and contains prospectively collected, quality controlled dataset based on the Standardised Audit of Hip Fractures in Europe (SAHFE) process [17].

The NUH renal AKI database is the largest single centre AKI database in the United Kingdom. It was developed in parallel with the Nottingham AKI e-alert, an automated real-time electronic alerting system that uses algorithms based on internationally recognised SCr-based definitions of AKI [18]. The AKI database includes all adult patients admitted overnight to Nottingham University Hospitals.

Data were extracted from the Hip fracture and AKI databases in accordance with Caldicott principles. We identified completed hospital episodes between 01/04/2007 and 31/03/2011 where a patient was admitted following hip fracture and where sufficient serial serum creatinine measurements were available to allow identification and staging of AKI using the 2012 Kidney Diseases Improving Global Outcomes (KDIGO) classification [19]. The AKI database automatically excludes patients known to be receiving regular dialysis for end-stage renal disease and patients who die before an overnight admission.

The Nottingham Hip Fracture Score (NHFS) was calculated for all patients. The NHFS is essentially a weighted seven factor frailty score specific to hip fracture: Age; Cognitive function on admission (Abbreviated Mental Test Score [20] <7); not living at home; sex (male); haemoglobin < 100 g L^-1^; previous malignancy; >1 comorbidity (stroke/transient ischaemic attack; cardiovascular disease; diabetes; previously diagnosed renal disease. It has previously been shown to predict 30-day post hip fracture mortality in the UK [21, 22] and internationally [23].

Basic patient characteristics recorded included: age; gender; ethnicity; and pre-admission renal function.

### Definitions and calculation of kidney dysfunction

#### Acute Kidney Injury (AKI)

For AKI staging, two serum creatinine (SCr) values are required. The renal database algorithm uses pre-admission baseline SCr (defined as the average Scr from all values measured 7-365 days pre hospitalisation), and compares this with first in-hospital measured Scr (AKI stage at admission). If a pre admission baseline is not available then an estimated Scr (eSCr) is calculated assuming a theoretical estimated glomerular filtration rate (eGFR) of 75 ml/min/1.73 m^2^ and using backward calculation of the Modification of Diet and Renal Disease (MDRD) [24] equation, as recommended by KDIGO. Staging of AKI is revised throughout the admission after each new SCr result and the overall worst AKI stage is recorded as the final AKI stage for the hospital episode. Ideally urine output criteria for AKI definition would be used in addition to SCr criteria but these data are not routinely recorded electronically and urine output measurement is not reliable in non-catheterised or incontinent patients (Table 1).

**Table 1:** KDIGO definition and staging of Acute Kidney Injury

Stage 1	Increase in Scr of 1.5 to 1.99 times baseline OR ≥ 0.3 mg/dl (≥26.5 μmol/l) increase.
Stage 2	Increase of between 2 and 2.99 times baseline
Stage 3	Increase of 3 times or more baseline OR increase in Scr to ≥ 4.0 mg/dl (≥353.6 μmol/l) after previous stage 1 OR initiation of renal replacement therapy (a retrospective staging).

*KDIGO* Kidney Disease | Improving Global Outcomes

*SCr* Serum creatinine

#### Chronic kidney disease (CKD)

CKD stage was calculated in patients with an actual baseline and a post discharge SCr. Pre-admission CKD stage was calculated using mean pre admission SCr, age, gender and ethnicity. Post-discharge CKD was calculated using mean post-discharge SCr (measured between 90 and 365 days following discharge), age, gender and ethnicity.

### Post-operative outcomes

Data were retrieved for: length of acute hospital stay; time to death (in hospital and post-discharge); postoperative admission to critical care; renal recovery (percentage change comparing 90-365 day post discharge SCr and pre-admission known baseline) and dialysis dependence (defined as attending dialysis clinic 90-365 days post discharge).

A minimum 90 day time frame for renal recovery is used to assess renal outcome post discharge, in accordance with the Kidney Disease Outcomes Quality Initiative guidelines which defines CKD as a persistent decline in kidney function lasting > 90 days [25], otherwise patients are defined as recovered renal function.

### Statistical methods

Data were analysed using R statistical package [26]. Continuous variables are presented as mean (95% confidence interval) or median [inter-quartile range] as appropriate. Categorical variables are described as proportions of the cohort. Student’s *t*-test was used to compare parametric data and the Mann-Whitney *U*-test was used to compare non-parametric data. Group comparisons were by either two-by-two contingency table with Chi-squared analysis, Kruskal-Wallis or ANOVA for multiple groups. Two-tailed *P*-values were calculated and a value of less than 0.05 was considered significant.

#### Modelling and prediction

Multivariate analysis of factors predictive of development of AKI was performed.

Logistic regression was conducted using development of AKI as the outcome and using all predictors with p < 0.2 on univariate analysis. Backwards elimination was used to optimise Akaike’s Information Criterion. Sensitivity analyses of this modelling were conducted: using age as a continuous rather than a categorical variable; and imputing CKD stage 2 for those patients without a measured pre-admission SCr.

Hazard ratios (HR) for mortality, with associated 95% confidence intervals (CI) were derived from Cox proportional hazard models stratified by AKI severity (using no AKI as the reference) or pre-admission eGFR (average for 7-365 days pre admission) group (using eGFR >60 ml/min/1.73 m^2^ as the reference) including age and sex as co-variates. Analyses were repeated using imputed CKD stage 2 (eGFR >60 ml/min/1.73 m^2^) for all those patients without a baseline SCr.

#### Predictive modelling

The full dataset was split at random [27] into training and testing subsets (ratio 0.66:0.34) using development of AKI as a sampling variable. Predictive risk scoring models were created using logistic regression modelling of the training dataset [28] using the predictors identified in the association modelling. Models were created using only patients with known pre-admission SCr and using imputed CKD stage 2 (eGFR >60 ml/min/1.73 m^2^) for all those patients without a baseline SCr. Model performance was assessed using Receiver Operating Characteristic (ROC) curves (ROC) (discrimination) and Hosmer-Lemeshow tests (calibration). Confidence intervals for area under the ROC curve (AUROC) were calculated using DeLong’s method [29] and confidence intervals for test performance measures were calculated using Wilson’s method [30] and Koopman’s method [31] (Likelihood ratios). The modelling is reported in accordance with the TRIPOD statement [32].

A simplified score was created based on the predictive modelling and assessed for discrimination and calibration on the testing subset as above.

## Results

We identified 2959 completed hospital episodes between 01/04/2007 and 31/03/2011, where a patient was admitted for hip fracture and sufficient serial Scr measurements were collected to allow presence or absence of AKI to be defined. The revised NHFS (11) was available in 96.2% (2848/2959). A valid SCr from the time period 7-365 days before admission was available for 2044/2848 patients (71.8%). Median (IQR [range]) follow-up was 691 (215 - 1119 [1 -2151]) days.

The distribution of NHFS was very similar to those previously reported [15, 16, 20, 33] with median (IQR) [range] of 4 (3-6) [0-10]. Of the 2848 individual hospital episodes, 683 patients (24%) developed AKI. These were divided into stage 1 (483/683 patients 70.7%), stage 2 (136/683 19.8%), and stage 3 (64/683 9.4%). Four patients required renal replacement therapy for AKI (1 from stage 2 and 3 from stage 3).

Tables 2 and 3 details patient characteristics and outcomes. Patients who developed AKI were older than those who did not (85 [78.6 to 89.8] vs 82.6 [75.2 to 87.9] years, *p* < 0.0001). This was true for men (83.8 [74.9 to 88.5] vs 78.3 [66.5 to 85.7], *p* < 0.0001) and women (86.0 [80.1 to 90.1] vs 83.8 [77.1 to 88.6] years, *p* < 0.0001). Patients with AKI were more likely to be male than those who were not (31.3% vs. 26%, *p* < 0.001). Length of stay was 15 [11 to 23] days for the no AKI group and 19 [13 to 31] days in those with AKI (*p* < 0.0001). Use of a critical care bed post-operatively increased with increasing stage of AKI.

**Table 2:** Characteristics and outcomes of included participants according to Acute Kidney Injury stage

Characteristics *Median [IQR]* *Number (proportion %)*	No AKI 2165	All AKI 683	Stage 1 483	Stage 2 135	Stage 3 64	*P* value *No AKI vs. All AKI*
Age, years	82.6 [75.2 to 87.9]	85.0 [78.6 to 89.8]	85.5 [78.6 to 89.9]	84.3 [78.5 to 89.6]	83.9 [77.4 to 88.9]	<0.001
Male	562 (26.0)	214 (31.3)	154 (31.9)	38 (27.9)	22 (34.4)	<0.001
Number (%) with known baseline SCr	1587 (73.3)	457 (66.9)	342 (70.8)	75 (55.0)	40 (62.5)	0.001
Baseline eGFR mls/min/1.73 m^2^	64 [50 to 77]	57 [43 to 74]	57 [42 to 72]	63 [48 to 72]	58 [37 to 81]	<0.001
CKD stage > =3	658/1587 (41.5)	243/457 (53.1)	186/342 (54.3)	36/75 (48)	21/40 (52.5)	<0.001
Diabetes	266 (12.3)	127 (18.6)	84 (17.4)	28 (20.7)	15 (23.4)	<0.0001
Previous Stroke or TIA	271 (11.9)	104 (15.2)	72 (14.9)	19 (14.1)	13 (20.3)	0.069
Cardiovascular disease	1157 (52.8)	438 (64.1)	301 (62.3)	92 (68.1)	45 (70.3)	<0.0001
Greater than one defined co-morbidity	658 (30.3)	301 (44.1)	205 (42.4)	61 (45.2)	35 (54.7)	<0.0001
Admission AMTS <7	743 (34.3)	244 (35.7)	171 (35.4)	49 (36.3)	24 (37.5)	0.5
Admission haemoglobin < 100 g L^-1^	154 (7.1)	61 (8.9)	49 (10.1)	8 (5.8)	4 (6.3)	0.13
Not admitted from home	441 (20.3)	90 (18.6)	32 (23.5)	9 (14)	131 (19.2)	0.51
Nottingham Hip Fracture Score	4 [3–5]	5 [4–6]	5 [4–6]	5 [4–6]	5 [4–6]	<0.0001
Nottingham Hip Fracture Score > = 4	1544 (71.3)	583 (85.4)	414 (83.1)	113 (83.1)	56 (87.5)	<0.0001
Nottingham Hip Fracture Score > = 5	1044 (48.2)	417 (61.1)	302 (62.5)	75 (66.4)	40 (62.5)	<0.001

*AKI* Acute Kidney Injury, *AMTS* Abbreviated mental test score

*eGFR* estimated glomerular filtration rate, *SCr* Serum creatinine

*CKD* Chronic kidney disease

**Table 3:** Outcomes of included participants according to Acute Kidney Injury stage

Characteristics *Median [IQR]* *Number (proportion %)*	No AKI 2165	All AKI 683	Stage 1 483	Stage 2 135	Stage 3 64	*P* value *No AKI vs. All AKI*
Length of Stay, days	15 [11–23]	19.1 [13–31]	19 [13–31]	20 [12.3 to 32.5]	20 [12–32]	<0.001
Part stay in critical care	40 (1.8)	41 (6.0)	16 (3.3)	11 (8.1)	14 (21.9)	<0.001
Proportion admitted multiple times, all 4 yrs.	1378 (63.6)	401 (58.7)	301 (62.3)	71 (52.2)	29 (45.3)	0.020
In patient deaths	134 (6.2)	169 (24.7)	100 (20.7)	39 (28.7)	30 (46.9)	<0.001
30-day mortality	139 (6.4)	131 (19.2)	79 (16.4)	27 (19.9)	25 (39.1)	<0.001
1 year mortality	534 (24.7)	305 (44.7)	203 (42.0)	61 (44.9)	41 (64.1)	<0.001

**Fig. 1:**
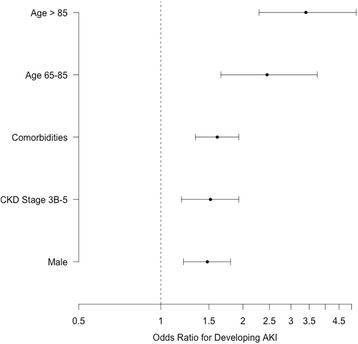
Multivariate odds ratios of developing AKI following admission with hip fracture

Table 2 details the univariate analysis of potential risk factors for development of AKI. Multivariate analysis with optimisation of AIC showed age, pre-existing CKD, sex and two or more co-morbidities to be independently predictive of development of AKI (Table 4, Fig. 1).

**Table 4:** Multivariate risk factors for development of Acute kidney injury during admission

	Coefficients	OR (95% CI)	*p*
Intercept	-2.476		<0.0001
Male sex	0.390	1.48 (1.21 to 1.80)	0.0004
CKD stage 3B-5	0.417	1.52 (1.19 to 1.93)	0.0008
Age 65 – 85	0.896	2.45 (1.66 to 3.74)	<0.0001
Age > 85	1.223	3.40 (2.29 to 5.20)	<0.0001
Two or more comorbidities	0.475	1.61 (1.34 to 1.93)	<0.0001

Patients without known Scr were imputed to CKD stage 2. The results for all predictors are almost identical if age is used as a continuous variable (OR 1.033 (1.024 to 1.043), *p* < 0.0001)

*AKI* Acute Kidney Injury

*CKD* Chronic kidney disease

*SCr* Serum creatinine

### Pre admission chronic kidney disease

The risk of developing AKI, and its severity, were associated with pre-admission SCr and eGFR. For the 2044 patients with a documented pre-admission SCr, Table 4 shows the inverse relationship between pre-admission eGFR, incidence and severity of AKI and 30-day mortality. In the no AKI group 26.7% (578/2165) did not have a pre-admission SCr available, whilst in the AKI group (all stages) it was 33% (226/683). For each stage of AKI the proportions without a known pre- admission SCr were: stage 1 29.2% (141/483), stage 2 44.4% (60/135) and stage 3 37.5% (24/64).

Pre-admission baseline eGFR (n = 1946) was inversely associated with in-hospital mortality within the whole cohort: eGFR <30 ml/min/1.73 m^2^ – 32.9% in-hospital mortality (25/76); eGFR 30-60 ml/min/1.73 m^2^ – 12.4% (98/727) and eGFR >60 ml/min/1.73 m^2^ - 10.4% (119/1143).

### Survival following hip fracture

**Fig. 2:**
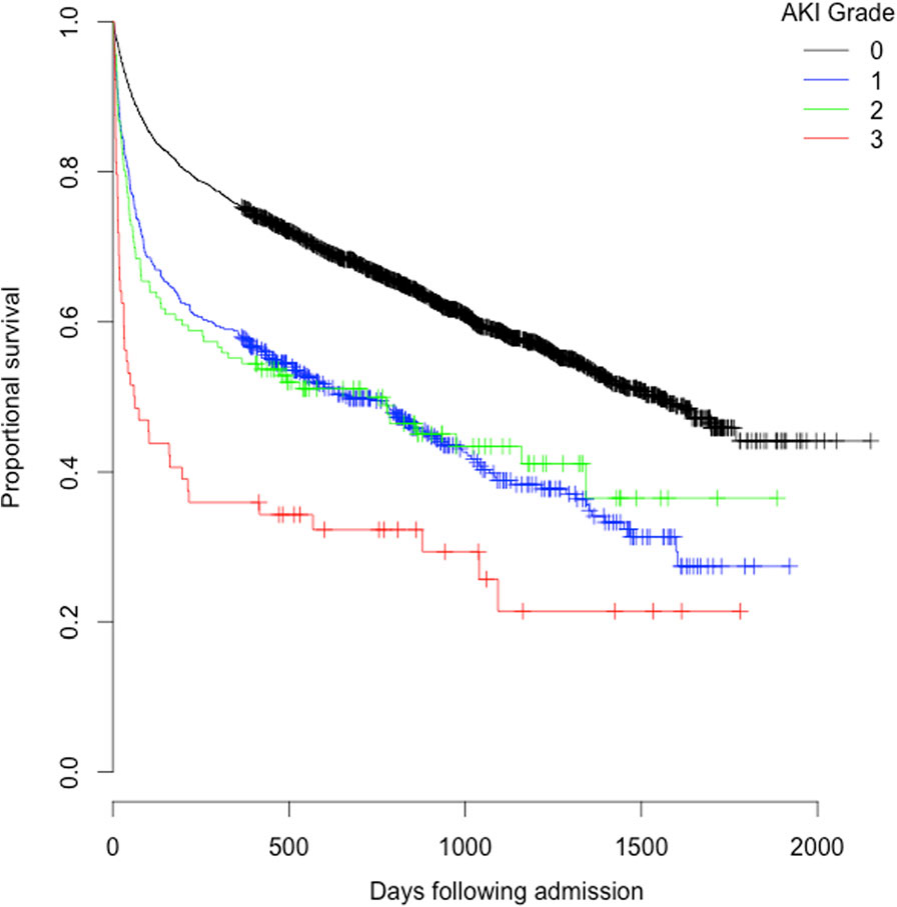
Kaplan Meier plot of cumulative probability of mortality day adjusted for age and gender and stratified for AKI severity

**Fig. 3:**
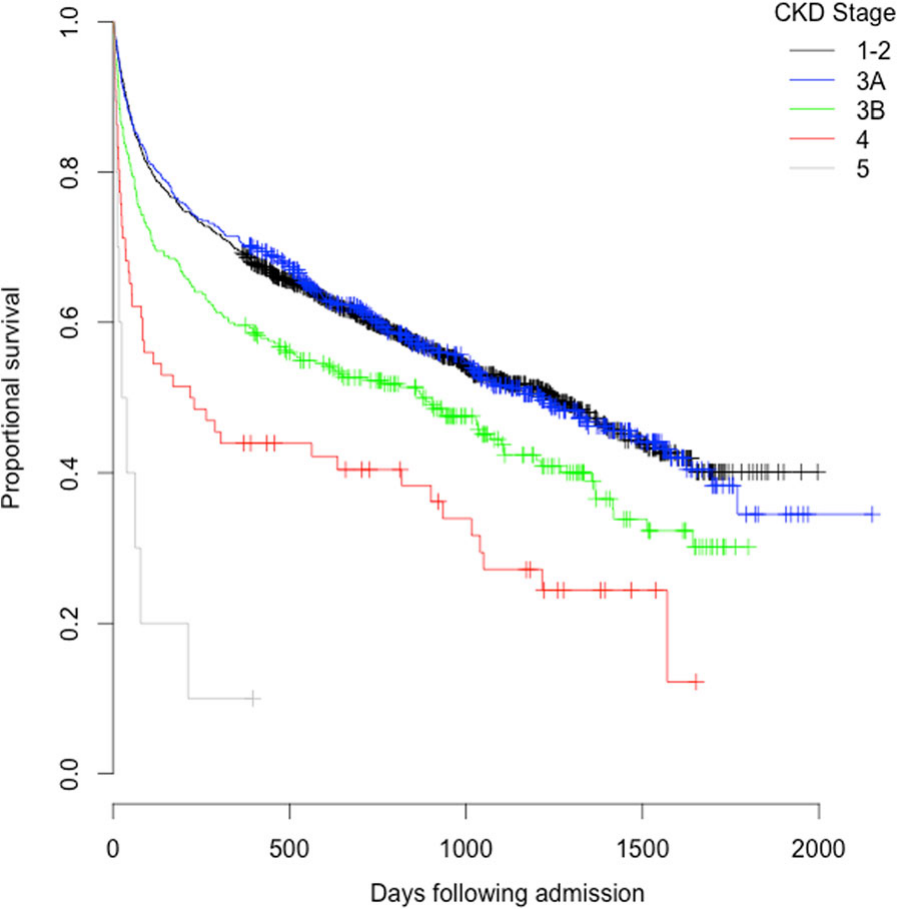
Kaplan Meier plot of cumulative probability of mortality day adjusted for age and gender and stratified for CKD severity

Mortality as an inpatient, 30-days and one year was increased for those patients who developed AKI and those with pre-existing chronic kidney disease (Tables 3 and 5; Figs. 2 and 3).

**Table 5:** Acute kidney injury and mortality according to pre-admission estimated glomerular filtration rate

Pre admission creatinine eGFR grouping	Number	AKI stage during admission Number (% of CKD group)					Mortality Number (%)	
		No AKI	Stage 1	Stage 2	Stage 3	All AKI	30 Day	One year
<15 ml/min/1.73 m^2^	10	1 (10)	3 (30)	0 (0)	6 (60)	9 (90)	5 (50)	9 (90)
15-29 ml/min/1.73 m^2^	66	36 (54.5)	25 (37.9)	4 (6.1)	1 (1.5)	30 (45.4)	19 (28.8)	37 (56.1)
30-44 ml/min/1.73 m^2^	292	202 (69.2)	72 (21.1)	11 (14.7)	7 (17.5)	90 (19.7)	46 (15.8)	118 (40.4)
45-59 ml/min/1.73 m^2^	533	419 (26.4)	86 (25.1)	21 (28.0)	7 (17.5)	114 (25.0)	50 (9.4)	157 (29.4)
>60 ml/min/1.73 m^2^	1143	929 (58.5)	156 (45.6)	39 (52.0)	19 (47.5)	214 (46.8)	97 (8.5)	350 (30.1)
Total	2044	1587 (100)	342 (100)	75 (100)	40 (100)	457 (100)	217 (10.6)	671 (32.8)

*AKI* Acute Kidney Injury

*eGFR* estimated glomerular filtration rate

*CKD* Chronic kidney disease

**Fig. 4:**
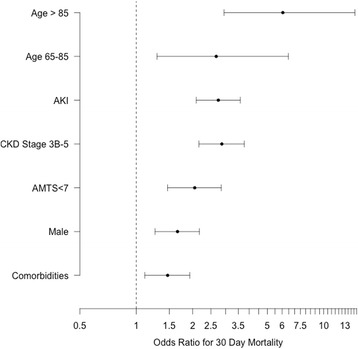
Multivariate odds ratios of 30-day mortality following admission with hip fracture

Multivariate analysis identified age (continuous and categorised), male sex, pre-existing CKD > =3B and AMTS <7 as independent risk factors for 30-day mortality (Table 6). The results were trivially different whether imputed or unknown CKD stage was used (Fig. 4).

**Table 6:** Risk factors for thirty-day mortality identified using multivariate logistic regression

	Coefficient	OR (95% CI)	*p*
Intercept	-3.786		<0.0001
Male sex	1.050	2.86 (2.16 to 3.77)	<0.0001
CKD stage > =3B	0.717	2.05 (1.47 to 2.84)	<0.0001
Age 65 - 85	0.981	2.67 (1.29 to 6.48)	0.0159
Age >85	1.801	6.05 (2.94 to 14.66)	<0.0001
AMTS <7	0.505	1.66 (1.26 to 2.17)	0.0002
AKI during admission	1.008	2.74 (2.09 to 3.59)	<0.0001
Two or more comorbidities	0.383	1.47 (1.11 to 1.93)	0.0067

Patients with no known baseline eGFR were assigned to CKD Stage 2. The results for all predictors are almost identical if age is used as a continuous variable (OR 1.069 (1.051 to 1.087); *p* <0.0001)

*AKI* Acute Kidney Injury

*eGFR* estimated glomerular filtration rate

*CKD* Chronic kidney disease

*AMTS* Abbreviated mental test score

Cox proportional hazards modelling including all the elements of the Nottingham Hip Fracture Score, CKD stage and AKI grade identified age (65-85 years 2.4 (1.8-3.3); >85 years 4.0 (3.0 – 5.4)), male sex (, all AKI grades, AMTS <7, residential status (all *p* < 0.0001), haemoglobin < 100 g L^-1^, (*p* = 0.0003), CKD stages > =3B (*p* = 0.003) and >1 comorbidity (*p* = 0.0003) as independent risk factors (Table 7).

**Table 7:** Hazard ratios for mortality using Cox proportional hazards modelling

	Hazard Ratio (95% CI)	*p*
Age 65 - 85	2.4 (1.8 to 3.3)	<0.0001
Age >85	4.0 (3.0 to 5.4)	<0.0001
Male sex	1.8 (1.6 to 2.1)	<0.0001
AMTS <7	1.6 (1.4 to 1.8)	<0.0001
Not admitted from home	1.6 (1.4 to 1.8)	<0.0001
Admission haemoglobin < 100 g L^-1^	1.4 (1.2 to 1.8)	0.0003
Two or more comorbidities	1.5 (1.3 to 16)	<0.0001
Any AKI	1.6 (1.4 to 1.8)	<0.0001
CKD stage > =3B	2.05 (1.47 to 2.84)	<0.0001

### Survivors and renal outcome

Of the 2545 survivors to hospital discharge, 48.2% (1227) had both a pre and post admission eGFR available. Incidence of any stage of AKI was 17.6% (216/1227) in this group.

Pre-admission and post-discharge eGFR were correlated for both no AKI and AKI groups (r^2^ 0.79 and r^2^ 0.82, *P* < 0.001 respectively). There was a statistically but not clinically significant improvement in renal function following discharge in the no AKI group (*n* = 1011) (63 [50 to 77] ml/min/1.73 m^2^ vs. 67 (53 to 83) ml/min/1.73 m^2^ (*p* = 0.0002). There was no change in the AKI group (*n* = 216) (59 [47 to 77] l/min/1.73 m^2^ vs 60 [48 to 77] ml/min/1.73 m^2^ (*p* = 0.48).

### Risk prediction models

Two risk prediction models were developed using the training set and the previously identified predictors. The patient characteristics and outcomes of the two groups were similar (Tables 8 and 9).

**Table 8:** Comparison of training and testing datasets – patient characteristics

Characteristics *Median [IQR]* *Number (proportion %)*	Training set (*N* = 1880)	Testing set (*N* = 968)
Age, years	82.9 [75.5 to 88.3]	83.7 [76.8 to 88.6]
Male	534 (28.4)	242 (25.0)
Number (%) with known baseline SCr	1352 (71.9)	692 (71.5)
Baseline eGFR mls/min/1.73 m^2^	63 [50 to 78]	61 [48 to 75]
CKD stage > =3	583 (43.1)	318 (46.0)
Diabetes	261 (13.9)	132 (13.6)
Previous Stroke or TIA	234 (12.4)	131 (13.5)
Cardiovascular disease	1011 (53.8)	540 (55.8)
Greater than one defined co-morbidity	629 (33.5)	330 (34.1)
Admission AMTS <7	619 (32.9)	368 (38.0)
Admission haemoglobin < 100 g L^-1^	538 (28.6)	314 (32.4)
Not admitted from home	363 (19.3)	209 (21.6)
Nottingham Hip Fracture Score	4 [3–6]	5 [4–6]
Nottingham Hip Fracture Score > = 4	1389 (73.9)	738 (76.2)
Nottingham Hip Fracture Score > = 5	908 (48.3)	513 (53.0)

**Table 9:** Comparison of training and testing datasets – patient characteristics

Characteristics *Median [IQR]* *Number (proportion %)*	Training set (*N* = 1880)	Testing set (*N* = 968)
Length of Stay, days	16 [12–25]	15 [11–24]
Part stay in critical care	54 (2.9)	26 (2.7)
Proportion admitted multiple times, all 4 yrs.	1161 (61.8)	618 (63.8)
In patient deaths	192 (10.2)	111 (11.5)
30-day mortality	164 (8.7)	106 (11.0)
1 year mortality	533 (28.4)	306 (31.6)
AKI 0	1429 (76.0)	736 (76.0)
AKI 1	320 (17.0)	163 (16.8)
AKI 2	85 (4.5)	51 (5.3)
AKI 3	46 (2.4)	18 (1.9)

Model 1: Age (<65, 65-85, >85), CKD stage (3B-5), two or more comorbidities, sex (male)

Model 2: Age (continuous), CKD stage (3B-5), two or more comorbidities, sex (male)

A third model was tested using a simplified scoring system based on Model 1 (Table 10). The predicted risk from the new score is calculated as:

**Table 10:** Prediction models characteristics

		Model 1	Model 2	Nottingham Hip Fracture – Risk Score for Kidney Injury (NH-RISK)
Coefficients				
	Intercept	-2.264	-3.78	-2.264
	Male sex	0.358	0.370	1
	CKD stage 3B-5	0.266	0.216	1
	Age (year)	-	0.028	
	Age 65 – 85	0.705	-	3
	Age > 85	1.023	-	4
	Two or more comorbidities	0.518	0.521	2

*CKD* Chronic kidney disease

$$ \mathrm{Predicted}\ \mathrm{risk} = 1/\left(1 + {\mathrm{e}}^{\left(2.264\ \hbox{--}\ \left[\mathrm{N}\mathrm{H}\hbox{-} \mathrm{RISK}/4\right]\right)}\right) $$

Where NH-RISK is the sum of the individual scores

**Fig. 5:**
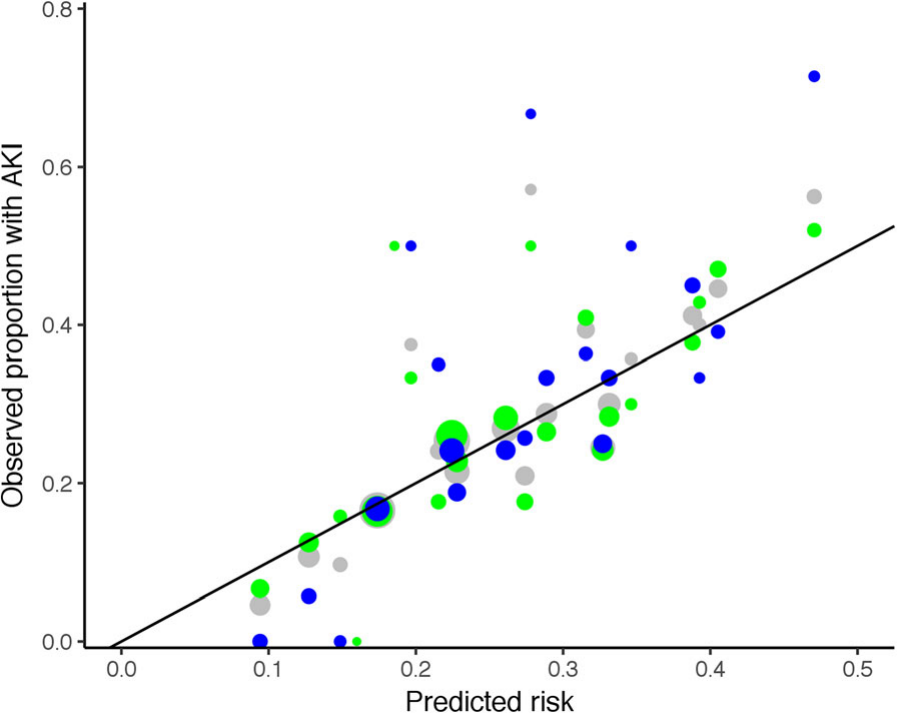
Calibration plot for model 1. Data for the full dataset are shown in grey, for the training set in green and the testing set in blue. The area of the points is proportional to the number of cases in each group. The line represents the line of equality, where observed and predicted outcomes are equal

**Fig. 6:**
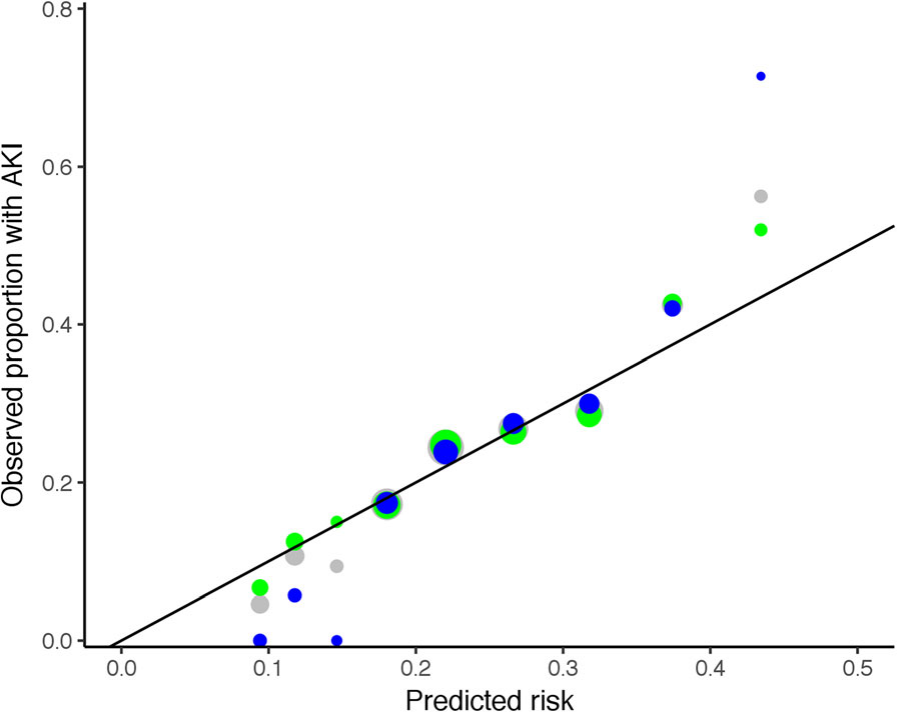
Calibration plot for the Nottingham Hip Fracture – Risk Score for Kidney Injury (NH-RISK). Data for the full dataset are shown in grey, for the training set in green and the testing set in blue. The area of the points is proportional to the number of cases in each group. The line represents the line of equality, where observed and predicted outcomes are equal

Areas under the ROC curves were 0.63 (0.59 to 0.67), 0.65 (0.61 to 0.69) and 0.63 (0.60 to 0.67) respectively. Calibration was adequate for all three models (*p* = 0.464, 0.438 and 0.847) (Table 11; Figs. 5 and 6).

**Table 11:** Calibration of the Nottingham Hip Fracture – Risk Score for Kidney Injury (NH-RISK)

NH-RISK score	Predicted risk	Observed rate of AKI (number/group size)		
		Full set	Training set	Test set
0	0.09	0.046 (5/109)	0.067 (5/75)	0 (0/34)
1	0.12	0.107 (14/131)	0.125 (12/96)	0.057 (2/35)
2	0.15	0.094 (3/32)	0.15 (3/20)	0 (0/12)
3	0.18	0.172 (102/592)	0.171 (67/391)	0.174 (35/201)
4	0.22	0.244 (206/843)	0.247 (137/554)	0.239 (69/289)
5	0.27	0.268 (134/500)	0.264 (87/329)	0.275 (47/171)
6	0.32	0.291 (125/430)	0.286 (80/280)	0.3 (45/150)
7	0.37	0.425 (76/179)	0.427 (47/110)	0.42 (29/69)
8	0.43	0.563 (18/32)	0.52 (13/25)	0.714 (5/7)

## Discussion

Our main findings are: first, AKI occurs in 1 in 4 hip fracture patients; second, mortality risk and acute length of stay increase with increasing severity of AKI; third, male sex, older age, pre-admission CKD and having more than one comorbidity are independent risk factors for AKI; and lastly, mortality risk post hip fracture is increased if pre-injury eGFR is <30 ml/min/1.73 m^2^.

The incidence of AKI in this large hip fracture population was 24%. This is greater than has previously been reported and is around twice as common as in the general surgical population [34]. White and colleagues [10] reported an incidence of 36.1% of patients whose estimated GFR *on admission* was < 60 ml/min/1.73 m^2^, but were unable to comment on whether this was acute, chronic or both. Previous studies of AKI following hip fracture have used considerably smaller sample sizes (<200) and have reported AKI incidence at around 16% [11] using either Acute Kidney Injury Network (AKIN) [25] or RIFLE [35] classifications of AKI. Until the publication of the KDIGO guidelines in 2012 this has been a major source of inconsistency in the classification and reporting of AKI incidence. This study is the first to use this validated classification and uses a large population suggesting that incidence has been under-estimated previously. Increasing age and male gender have previously been reported as being associated with poorer outcomes after hip fracture [30, 36]. A large meta-analysis incorporating 94 studies reported advancing age and male gender to be the two strongest predictors of mortality in hip fracture surgery patients [37]. We add to this knowledge with another large study. Both chronic kidney disease (CKD) and AKI are associated with greater and earlier mortality [38–41]. Patients with pre-admission eGFR <30 ml/min/1.73 m^2^ had a relative risk of developing AKI of 2.4 compared with those with eGFR >30 ml/min/1.73 m^2^, supporting data that suggest pre-existing CKD increases risk of AKI [34, 42, 43]. AKI was associated with an increase in mortality. Of note, stage 1 and 2 AKI appear to carry the same intermediate increase in risk. This would support the assertion that ‘mild’ derangement of renal function, which is commonly seen (around 1 in 5) in this elderly population, is not benign. All mortality rates (in-hospital, 30 day, 90 day and 1 year) increased with increasing severity of AKI and with lower pre-admission renal function, demonstrating the importance of identifying both AKI and CKD in the elderly.

This is a single centre study so our data may not be replicated elsewhere, and the inherent weaknesses of logistic regression modelling have been discussed by others [44]. However, outcomes following hip fracture in Nottingham are similar to elsewhere [16, 45] and the demographics of our hip fracture population are similar to the rest of the UK. The rates of CKD and AKI are similar to previous studies, bearing in mind the differences in methodology. The strength of our study includes the use of two large prospectively collected clinical databases with almost complete data capture for the four-year period. We were able to estimate pre-injury renal function in 72% of participants, which allows us to diagnose AKI on admission blood tests. We were also able to determine post-discharge renal function. The use of calculated pre-admission SCr, assuming normal GFR where no documented result is available, may result in over-estimation of the incidence and stage of AKI. Nevertheless, the data presented here demonstrate clinically relevant outcomes: the survival curves for Stage 1 and stage 2 AKI are essentially identical, and worse than those with no AKI. We have not been able to identify a highly discriminating combination of predictor variables – the AUROC was relatively poor at around 0.63. However, calibration is reasonable, suggesting that either the full models, or the simplified 9-point NH-RISK may be useful tools for classifying risk of AKI on admission. Accurate assessment of risk is widely perceived to be a driver to improved management, though direct evidence of changes in clinical care and outcomes is lacking. We intend to trial the use of the simplified scoring system as part of our hip fracture admission criteria, in the first instance simply to flag those patients identified as highest risk of AKI who may benefit from specific clinical interventions.

CKD is both a risk factor for fracture [38] and associated with poorer outcome. It is tempting to be nihilistic and ascribe CKD as a fixed predictor of poor outcome. However, AKI is potentially preventable in some patients, so should be viewed as a possible target for intervention. Simple interventions such as avoidance of nephrotoxic drugs and correction of hypotension and hypovolaemia are important, yet easily overlooked. NSAIDs are usually avoidable, at least during the period of risk for AKI. Similarly, there are often alternatives to nephrotoxic antibiotics such as gentamicin but, if considered the antimicrobial of choice, nephrotoxicity can be reduced with meticulous monitoring of blood levels. Angiotensin converting enzyme (ACE) inhibitors and angiotensin receptor blockers are commonly prescribed in the elderly and are not nephrotoxins *per se*. There is currently no clear evidence on when to discontinue these drug classes peri-operatively with respect to reducing risk of AKI. Current consensus suggests temporary discontinuation where AKI [14] or hypotension has developed. The risk of hypovolaemia may be reduced by limitation of starvation times and with intravenous fluid administration before and during surgery. Intraoperative stroke volume guided fluid studies are currently underpowered to demonstrate statistically significant changes in development of AKI [46, 47] and echocardiography studies [48] suggest that around 25% of patients are still hypovolaemic immediately prior to theatre. Patients arriving in theatre with urinary evidence of ‘dehydration’ [49] also have a worse outcome. Hypotension, relative or absolute, is very common intra-operatively, both with spinal and general anaesthesia [50].

Acute kidney injury is a costly disease with National Health Service (NHS) costs estimated to be between £434 million and £620 million per year [14]. The English hip fracture population is predicted to increase in size from around 70000 per year currently to around 90-100000 by 2033 [51]. The medical complexity of patients with hip fracture is also increasing in particular with increasing numbers of patients with cardiovascular and renal disease on admission [52]. The increased requirements for a critical care bed and longer hospital stay when AKI occurs add to this burden. Taken together this would suggest that hip fracture-related AKI is likely to have a significant and increasing financial burden in the near future.

## Conclusions

CKD and AKI are common findings in elderly hip fracture patients. Both are independently associated with poor outcome – mortality, critical care use and length of acute hospital stay. They are probably markers of chronic and acute comorbidity rather than the direct cause of death themselves. When available, pre-injury SCr should be obtained to facilitate early detection of AKI. The optimal approach to preventing AKI in this population is not yet known, but close monitoring of fluid balance, avoidance of nephrotoxic drugs, appropriate adjustment of renally excreted drugs and prompt treatment of sepsis would seem sensible in all peri-operative patients.

## Abbreviations

ACE: Angiotensin converting enzyme
AIC: Akaike’s Information Criterion
AKI: Acute Kidney Injury
AKIN: Acute Kidney Injury Network
AMTS: Abbreviated Mental Test Score
CKD: Chronic Kidney Disease
eGFR: estimated glomerular filtration rate (ml/min/1.73 m^2^)
IQR: Interquartile range
KDIGO: Kidney Diseases Improving Global Outcomes
MDRD: Modification of Diet and Renal Disease
NHFS: Nottingham Hip Fracture Score
NH-RISK: Nottingham Hip Fracture – Risk Score for Kidney Injury
NHS: National Health Service
NUH: Nottingham University Hospitals
RIFLE: Risk | Injury | Failure | Loss | End stage renal disease
ROC: Receiver Operating Characteristic
SAHFE: Standardised Audit of Hip Fractures in Europe (SAHFE)
SCr: Serum Creatinine
